# Risk factors associated with suicide clusters in Australian youth: Identifying who is at risk and the mechanisms associated with cluster membership

**DOI:** 10.1016/j.eclinm.2020.100631

**Published:** 2020-11-20

**Authors:** Nicole T.M. Hill, Matthew J. Spittal, Jane Pirkis, Michelle Torok, Jo Robinson

**Affiliations:** a Telethon Kids Institute, 15 Hospital Ave, Nedlands, Perth, WA 6008, Australia; b Orygen, Centre for Youth Mental Health, University of Melbourne, Melbourne, Australia; c Centre for Mental Health, Melbourne School of Population and Global Health, University of Melbourne, Melbourne, Australia; d Black Dog Institute, University of New South Wales, Sydney, Australia

## Abstract

**Background:**

It is unclear who is at risk of being involved in a suicide cluster and whether suicide clusters are influenced by the social transmission of suicidal behaviour, assortative relating, or a combination of both.

**Methods:**

Suicide clusters involving two or more young people were identified from the free text of electronic police and coroners reports in Australia's National Coronial Information System in a nationwide cross-sectional study. The duration of survival among exposed cases were estimated using time-to-event methods. The casewise concordance of demographic, social and clinical characteristics and circumstances of death were examined among index and exposed cases.

**Findings:**

We identified links between 117 young people (51 suicide clusters). 50% of young people died within 90 days of the index suicide. Individuals exposed to railway suicide had an 80% probability of dying by the same method. Those exposed to the suicide of a person aged 10–18 years had an 86% probability of being from the same age group. Young people had a 67% and 60% probability of sharing the same characteristics as the index suicide when the index suicide resided in a remote community or was of Aboriginal and Torres Strait Islander descent.

**Interpretation:**

Suicide clusters may be associated with both the social transmission of suicidal behaviour and assortative relating. Individuals who were close to the deceased should be provided with access to postvention support, particularly within the first 90 days of exposure to an index suicide.

**Funding:**

Australian Rotary Health, National Health and Medical Research Council.

**Research in context**

**Evidence before this study**

Young people are particularly susceptible to the phenomenon whereby exposure to the suicide of another increases a young person's own risk of suicidal behaviour. However it is not clear who is at risk of being involved in a suicide cluster, nor the extent to which suicide clusters are influenced by the social transmission of suicidal behaviour, or whether young people cluster together according to pre-existing characteristics (or risk factors) such as adverse mental health or substance misuse.

**Added value of this study**

Suicide clusters comprising social links between friends and acquaintances were identified using nationwide data of all young Australians, aged 10–24 years, who died by suicide over a ten-year period. The characteristics and risk factors associated with suicide clusters were identified, providing evidence of both the social transmission of suicidal behaviour and assortative relating among cluster members.

**Implications of all the available evidence**

Young people involved in suicide clusters do not necessarily share the same clinical risk factors associated with suicide in non-cluster members (e.g., adverse mental health or substance misuse). Interventions that prevent the spread of potentially harmful information about suicide among young people has the potential to avert further suicide deaths, particularly within in the first 90 days of exposure to the suicide of a friend or acquaintance. Together, these findings highlight a critical window of opportunity for the prevention of suicide clusters in young people who have been exposed to the suicide of another.

Alt-text: Unlabelled box

## 1 Introduction

Suicide is the second leading cause of death in young people aged 10–24 years, worldwide and accounts for approximately 150,000 preventable deaths in young people under age of 25, each year [1]. An individual's risk of suicide increases sharply during adolescence, as does the risk of being involved in a suicide cluster [2,3]. Population-based studies that define a suicide cluster in terms of a greater than expected number of suicides, occurring within a particular time and place, have shown that young people are two to four times more likely to be involved in a suicide cluster, relative to adults aged 25 years and above [4,5].

Studies of suicide clusters in community settings typically characterise suicide clusters on the basis of shared social links between two or more suicides in specific settings such as schools, inpatient units, and remote communities [6,7]. Clusters of this nature have been linked to prior exposure to suicide, a known risk factor for later suicide [8], and can lead to widespread speculation about the antecedents and circumstances of the deaths [9,10], prolonged grief [11] and heightened fear and anxiety among members of the community of the prospect of future suicides [2,9]. Together, these factors have been associated with further suicides in the community and have led to the development of cluster response guidelines [12], [13], [14] as well as sentinel surveillance systems for the detection of both self-harm and suicide clusters [15].

Multiple mechanisms have been put forth to explain how exposure to suicide may influence subsequent suicidal behaviour in others including social learning— the notion that young people learn from and model the behaviour they see in others; and descriptive norms, which describes the process where individuals are more likely to endorse suicidal behaviour as an acceptable option when they perceive it to be common [16,17]. Examples of the association between the social transmission of suicidal behaviour and suicide clusters has been reported following sensationalist newspaper reports of suicide [18], and following memorials (including those online) which glorified the deceased [9,10]. Social integration and regulation describes the influence that social structures and expectations have on an individual's behaviour and may be influenced by both exposure to suicide and the presence of pre-existing risk factors [17,24,25]. For example, in highly integrated communities, individuals who perceive themselves as different to may be particularly susceptible to suicide. Additionally the close social ties among members of a highly integrated community may facilitate the spread of information about suicide to vulnerable members of the community [25].

Another theoretical model, known as assortative relating, is based on the premise that individuals who are vulnerable to suicide may already cluster together according to pre-existing characteristics (or risk factors) such as adverse mental health or history of self-harm [19]. According to an assortative relating model, suicide clusters may emerge in response to simultaneous exposure to external pressures or stressful events among vulnerable peer groups [19]. An assortative relating hypothesis appears consistent with previous studies that have shown that young people tend to associate with peers who share similar risk factors such including levels of depression [20] and aggression [21] and is consistent with previous case-control studies of suicide clusters that report higher rates of prior substance misuse [22], self-harm behaviours [22], and pre-existing mental ill health [22,23] among young people involved in a suicide cluster, relative to living controls.

Systematic reviews examining the mechanisms of suicide clusters have suggested that both the social transmission of suicidal behaviour and assortative relating may operate to varying extents for suicide clusters in different communities [2,6]. However, direct comparisons of the mechanisms underlying suicide clusters have not yet been conducted using data from national suicide mortality registries. Together, the ecological design of many nationwide, population-based studies of suicide clusters means that it is not possible to determine whether members of a suicide cluster were exposed to suicide to one another, nor the extent to which members of a suicide cluster shared similar demographic, social, and clinical risk factors; both of which are critical for identifying who is at risk and identifying what mechanisms might be operating during a suicide cluster.

Identifying the risk factors and mechanisms that are associated with suicide clusters has the potential to better identify who is at risk of being involved in a suicide cluster and inform the development of targeted interventions designed to avert further suicide deaths. This is particularly important given recent evidence which suggests that the social links between cluster members differ significantly between different cluster events— suggesting that some, but not all suicide clusters, involve social links between cluster members [26]. Arguably, suicide clusters that are facilitated by the social transmission of suicidal behaviour may warrant different preventative approaches (e.g., strategies to prevent the spread of potentially harmful information such as the methods of suicide) than those which are associated with pre-existing risk factors (e.g., those which may be better address by upscaling or improving access to mental health services).

This study aimed to compare the demographic, social and clinical characteristics of pairs of young people in Australian suicide clusters and the circumstances of their deaths, in order to describe the risk factors associated with suicide clusters and to identify important characteristics of suicide clusters (e.g., the duration of risk associated with cluster membership). Additionally, we sought to identify the mechanisms underlying suicide clusters. We assumed that if assortative relating was operating, then the pairs of young people would share characteristics in common, and that if the social transmission of suicidal behaviour was operating then the circumstances surrounding their deaths would be similar.

## 2 Methods

### 2.1 Sample and case ascertainment

We conducted a retrospective cross-sectional analysis of young people who had died in a suicide cluster in Australia between 2006 and 2015 based on data recorded in the National Coronial Information System (NCIS). The NCIS is an online data storage and retrieval system that is used to monitor external causes of death in Australia [27,28]. Each case recorded in the NCIS includes a core data set that comprises basic demographic information (e.g., age, sex, Aboriginal and Torres Strait Islander status, employment status, student status, residential remoteness). Additional information on the circumstances surrounding each individual's death is available in the form of post-mortem autopsy and toxicology reports, as well as from the narrative text of police and coroner reports. Eligible cases were extracted from the NCIS database (https://www.ncis.org.au/) in April 2017 and an updated search for closed cases was conducted in June 2019. Data was extracted directly from narrative text between April 2017 and June 2019.

Suicide clusters were defined as two or more suicides occurring among young people aged 10–24 who shared social links as friends or acquaintances [13]. A case was eligible for inclusion in the analyses if: (1) the death occurred between 1 January 2006 and 31 December 2015; (2) the individual was aged 24 years or less at the time of death; (3) the case included at least one electronic police or coroner report that provided information on the demographic, social, clinical characteristics as well as the circumstances of death; (5) and the case was closed (fully investigated) by the coroner. We retrieved data on 3365 suicide cases from the NCIS. Of these, 338 cases (including all 245 cases from the state South Australia) were excluded due to the absence of information in the narrative text of police and coroner reports. This resulted in a total of 3027 suicide cases that were assessed for evidence of social links with other young people who had died by suicide.

### 2.2 Links between index and exposed cases

We labelled the first suicide case as the index case and labelled cases that were known to have been exposed to one or more index case as exposed cases. We used the following information to identify linked cases within a suicide cluster: (1) the narrative text referred to the first and last name of the person who previously died by suicide (the index case) or; (2) the narrative text referred to the date of death (e.g., month and year) and described at least one other characteristic that could identify the index case based on information included in the case records (e.g., the name of the school, the address, or the place of employment of the individual who died by suicide). No limits were placed on the duration between index and exposed cases other than that the suicides occurred during the ten-year study period. These cases formed the basis of the study data.

### 2.3 Characteristics and circumstances of death

Demographic, social, and clinical characteristics and the circumstances of death were extracted from the NCIS for each case. These included: age, sex, Aboriginal and Torres Strait Islander status, employment status, student status, residential remoteness, relative socioeconomic advantage and disadvantage, method of suicide, date of death and location of death. The location of residence, remoteness of residence, and socioeconomic advantage and disadvantage were aggregated at an area level, based on the Australian Statistical Geography Standard Statistical Areal Level 2 (SA2). We calculated the centroid (the geometric centre) of each SA2 and stored these data as longitude and latitude points for each eligible case in the NCIS and calculated the distance between the residence of index and exposed cases. Information on social and clinical characteristics were extracted by examining the narrative text in electronic police reports, coroner reports, autopsy reports, and toxicological findings using content analyses. Definitions used to identify the presence or absence of each variable are shown in Table 1. The first author (NTMH) identified characteristics a priori in consultation with the senior author (JR) and extracted these data into a proforma developed for this study.

**Table 1:** Table of definitions of included variables.

Variable	Definition and ascertainment	Variable
Age^a^	Determined by the NCIS core dataset	Demographic
Sex^a^	Determined by the NCIS core dataset	Demographic
Aboriginal or Torres Strait Island status^a^	Determined by the NCIS core dataset	Demographic
Student status^a^	Determined by the NCIS core dataset	Demographic
Employment status^a^	Determined by the NCIS core dataset	Demographic
Socioeconomic disadvantage (SEIFA) a	Determined by SA2 place of residence. The variable represents people who resided in regions that were identified in the bottom 20th percentile of socioeconomic disadvantage determined by the Australian Bureau of statistics Socio-economic index for advantage and disadvantage based on Statistical Area level 2.	Demographic
Remoteness^a^	Determined by SA2 place of residence. The variable represents people who resided in regions that were identified as remote or very remote in the NCIS.	Demographic
Distance between the residence of index and exposed cases^a^	Based on the geographic centroid of place of residence based on SA2 geography recorded in the NCIS core dataset.	Demographic
Parent separation^a^	Based on informant reports that the person's biological or adoptive parents were divorced or separated at the time of death or otherwise married or living in a de facto relationship. A definition of parent separation includes indirect evidence (e.g., narrative descriptions that indicate the young person lived with their mother).	Social
Living alone^a^	Based on informant reports or other documented evidence (e.g., police observation) that the person lived alone (e.g., the absence of roommates or other cohabitating household members).	Social
History of abuse and/or neglect^a^	Based on an informant report or other documented evidence (e.g., police statements) that the person had been sexually, physically or emotionally abused, or that the person had experienced neglect in their family home. Excludes informant reports of suspected abuse or neglect.	Social
Exposure to domestic violence^a^	Based on an informant report or documented police statements that the person had experienced violent or aggressive behaviour within the home. Includes exposure during childhood to domestic violence between parents.	Social
Relationship breakdown with an intimate partner (past 1 month) a	Based on an informant report that the person had a relationship breakdown with an intimate partner. Includes teenage boyfriend/girlfriend relationships and online relationships.	Social
Peer conflict (past 12 months) a	Based on an informant report that the person experienced relationship problems (e.g., arguments or physical confrontations) with their peers, or those in their friendship circle, in the 12 months prior to their death.	Social
Family conflict (past 12 months) a	Based on an informant report that the person had experienced relationship problems (e.g., arguments or physical confrontations) with their family members. Includes conflicts in the family environment that described the person had feelings that they were not able to meet family expectations.	Social
Diagnosed or undiagnosed mental ill health^a^	Based on documented medical evidence that the young person: (1) had a psychiatric diagnosis prior to their suicide; *OR* (2) the young person was being treated by a medical practitioner (e.g., GP, psychologist, psychiatrist) for mental-illness; *OR* (3) an informant reported that the young person was being treated for mental health symptoms *AND* results from the NCIS toxicology report described the presence of therapeutic levels of psychotropic medication (e.g., antidepressants) at or around the time of death; *OR* (4) the young person told an informant that they had mental health problems, but it was not otherwise verified by official records during the police or coroner investigation or evidence of therapeutic levels of psychotropic medication in the toxicology findings.	Clinical
Suicide attempt or self-harm (irrespective of intent) a	Based on an informant report or documented medical evidence that the person had made a suicide attempt or had self-harmed in the 12 months prior to their suicide. Includes presenting to healthcare services (e.g., ED) for a suicide attempt, informant descriptions of self-harm or suicide attempts, as well as indirect evidence from autopsy reports (e.g., the autopsy reported evidence of self-inflicted injuries or evidence of healing wounds from self-inflicted injuries such as abrasions to the wrist or consistent with cutting). Does not include informants reports of suspected self-harm or suicide attempt.	Clinical
Discharged from the Emergency Department (ED) for mental health symptoms^a^	Based on an informant report or documented medical evidence that the person presented to the ED prior to their suicide for reasons primarily related to mental health symptoms (including self-harm) and was discharged from the ED. Excludes presentations to the ED for reasons unrelated to mental health (e.g., suspected appendicitis) or informant reports of ED presentations for reasons unknown (e.g., may or may not have presented to the ED primarily because of mental health symptoms).	Clinical
Illicit substances detected in toxicology report^a^	Based on toxicology findings that indicated the presence of illicit substances (e.g., methamphetamine, cannabis, cocaine, MDMA) and/or their by-products demonstrating recent use of illicit substances. Includes substances of misuse (e.g., taurine from paint, petroleum from petrol).	Clinical
Binge Drinking^a^	Based on an informant report that the young person had been drinking excessively in the month prior to their death.	Clinical
Alcohol intoxication^b^	Based on toxicology findings that indicated a blood alcohol concentration >1500 mmol/l. Excludes alcohol readings from urine samples and toxicology reports that indicated that the alcohol concentration was likely to be influenced by post-mortem changes (e.g., decomposition).	Circumstances of suicide
Method of suicide b	Determined by coroner report and ICD-10 code for cause of death.	Circumstances of suicide
Suicide note b	Based on an informant report or police observation that the person had written a suicide note that indicated their intent to die. Includes goodbye text messages and emails sent by the deceased prior to their suicide.	Circumstances of suicide
Told someone they wanted to die b	Based on an informant report that the person expressed suicidal ideation or indicated that they wanted to take their own life or die. Includes written, verbal communication, text communication and email communication.	Circumstances of suicide
Communicated about suicide on social media b	Based on an informant report or evidence of online activity that showed the person posted content about suicide or expressed suicidal ideation on social media (e.g., posted a suicide note on Facebook, or shared a post that indicated they were suicidal).	Circumstances of suicide
Searched the internet for suicide methods b	Based on the police or coroner report that described evidence of internet queries about suicide or the person had visited a website that described methods of suicide.	Circumstances of suicide
Date of death	Based on the date (dd/mm/yyyy) of death. Date of death was determined by the coroner and recorded in the NCIS core dataset.	Circumstances of suicide

a Included in the analysis of assortative relating.

b Included in the analysis of imitative suicidal behaviour.

### 2.4 Data analysis

The distance between index and exposed cases was estimated by calculating the shortest possible distance between SA2 centroids using the geosphere package in R version 3.6.2. The geocode function of the ggmap package was used to identify the centroids and corresponding longitude and latitude geocoordinates of each SA2. Post hoc analyses were conducted to estimate the correlation between distance between index and exposed cases and the duration since exposure.

The duration of risk following exposure to an index suicide was determined using survival analysis. Survival time (defined as the time in days between the date of the suicide of the index case and the date of death of the exposed case) was estimated for each exposed case by subtracting the date of index death from the date of the exposed death. Kaplan-Meier survival curves were drawn using the survival and survminer packages in R 3.6.2.

The average annual percent change based on the date of the index death was calculated for both the number of suicide clusters and the number of cluster members, each year, over the 10 year study period.

The risk factors and characteristics associated with cluster membership was identified using casewise concordance under complete ascertainment [29] for the demographic, social and clinical characteristics and the circumstances of death among index and exposed cases (Table 1). The casewise concordance is the conditional probability that if one member of pair has the characteristic of interest, the other also has the same characteristic. In the present study, casewise concordance was defined as the probability that the exposed case had the characteristic of interest (e.g., male sex), given that the index case has the same characteristic. The probabilities are reported as percentages with corresponding 95% confidence intervals.

Due to power limitations, we excluded variables from the casewise concordance analyses if they were recorded in fewer than 5 index and exposed cases. Since any index case or exposed case could be linked to multiple cases (e.g., two exposed cases could be linked to the same index case) and later cases in clusters of three or more suicides could be included as both an index and exposed case (e.g., the second case in a cluster of three suicides might be exposed to the suicide of the first case but may also serve as an index case for the third case within the cluster), the assumption of statistical independence was not met. Therefore, to allow for correlations between index and exposed cases within we adjusted the standard errors for dependency within clusters.

The mechanisms underlying suicide clusters were operationalised by examining the circumstances of death and the demographic, social and clinical risk factors recorded in the NCIS. Specifically, evidence of the social transmission of behaviour was identified by examining the casewise concordance associated with the circumstances of death (Table 1). Evidence of assortative relating was operationalised by examining the probability that index and exposed cases shared the same demographic, social and clinical characteristics.

This study received full ethical approvals from the Justice Human Research Ethics Committee (CF/15/13188).

### 2.5 Role of the funding source

The funders of the study had no role in study design, data collection, data analysis, data interpretation, or writing of the report. The corresponding author had full access to all the data in the study and had final responsibility for the decision to submit for publication.

## 3 Results

### 3.1 Identification of suicide clusters

**Fig. 1:**
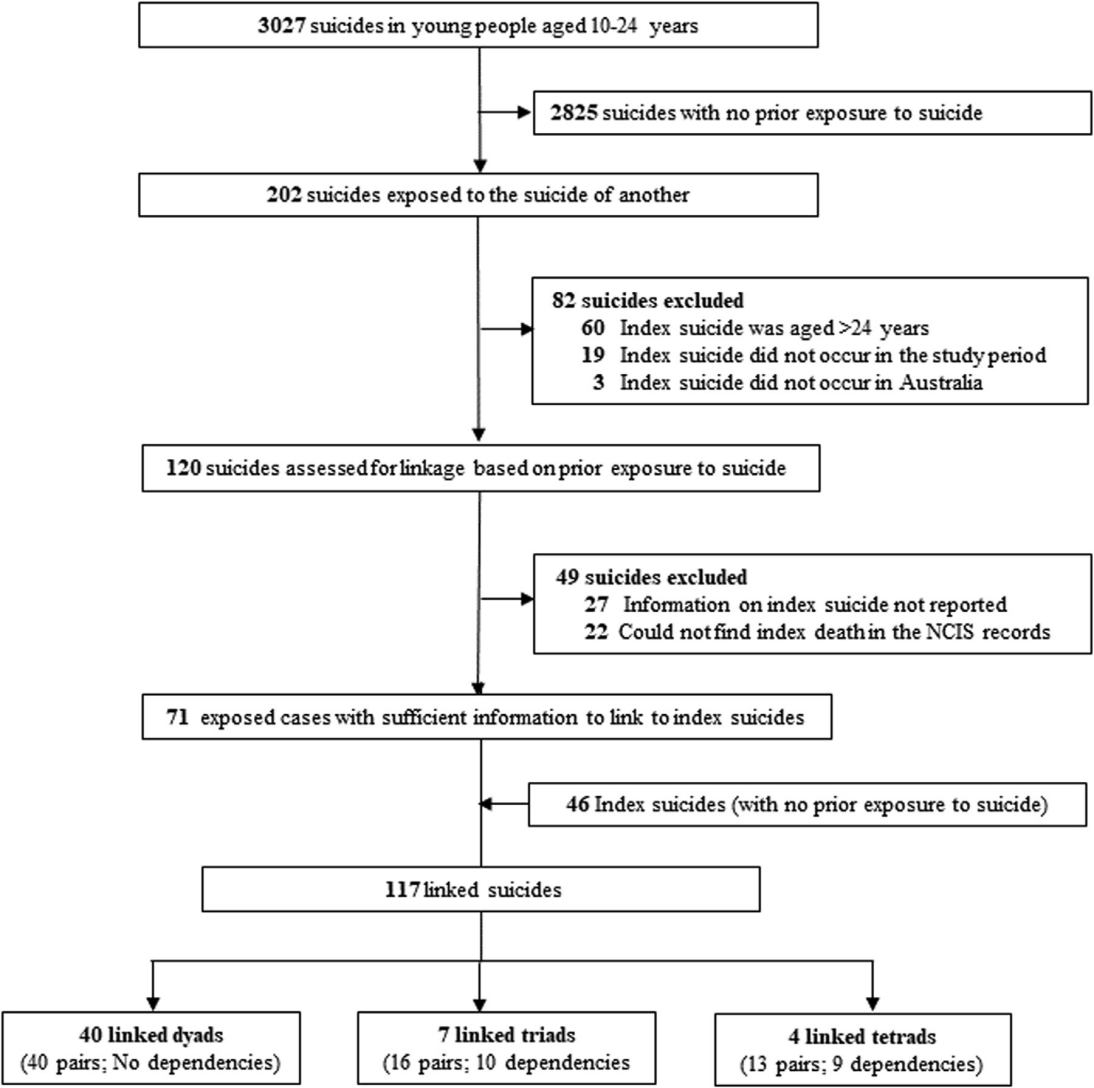
Flowchart of suicides and cluster-linked cases of young people aged 10–24 years who died by suicide in Australia in 2006–2015.

Overall, 120 exposed cases (young people who were exposed to the suicide of another young person) were manually inspected for linkage to an index suicide (Fig. 1). Of these, we found social links between 71 (59%) exposed cases that were linked to an additional 46 suicides, resulting 117 index and exposed cases that were included in the analyses. A total of 19 dependencies were identified (appendix table A1) resulting in 69 linked pairs across 51 suicide clusters. The suicide clusters comprised40 dyads (*N* = 80 suicides), 7 triads (*N* = 21 suicides) and 4 tetrads (*N* = 16 suicides, appendix table A1). We could not identify reliable links among 49 young people (48%) who were exposed to suicide during the study period. The characteristics of linked and missing cases are presented in (appendix table A2).

### 3.2 Characteristics of suicide clusters

**Fig. 2:**
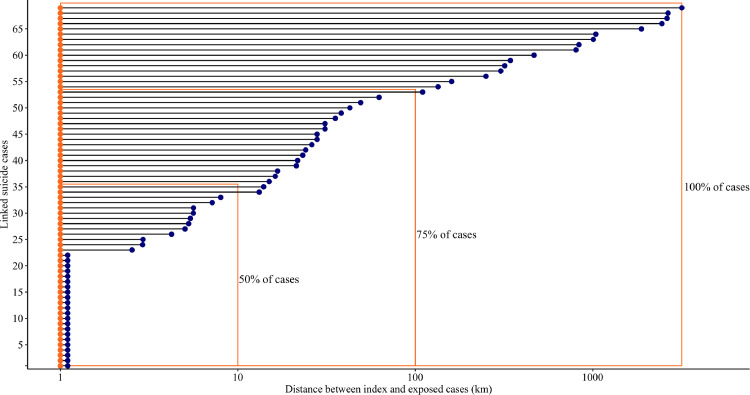
Geographic distribution of suicide clusters based on SA2 geography. Shows the spatial distribution in kilometres (km) between index and exposed cases who died in suicide clusters.

**Fig. 3:**
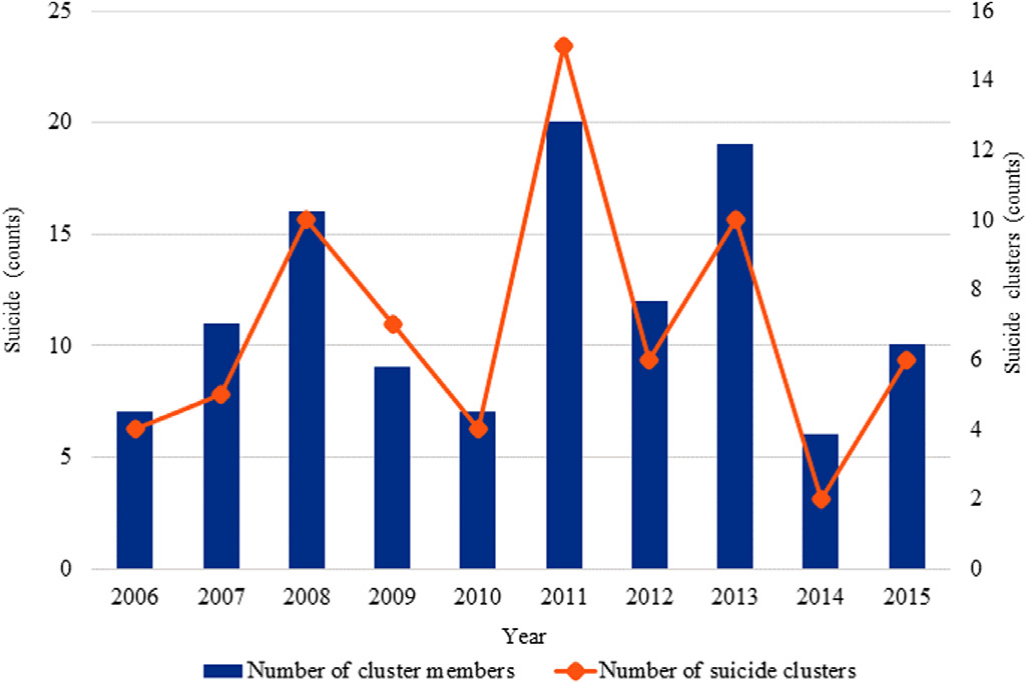
Number of incident suicide cluster events and cluster members by year (2006–2015).

**Fig. 4:**
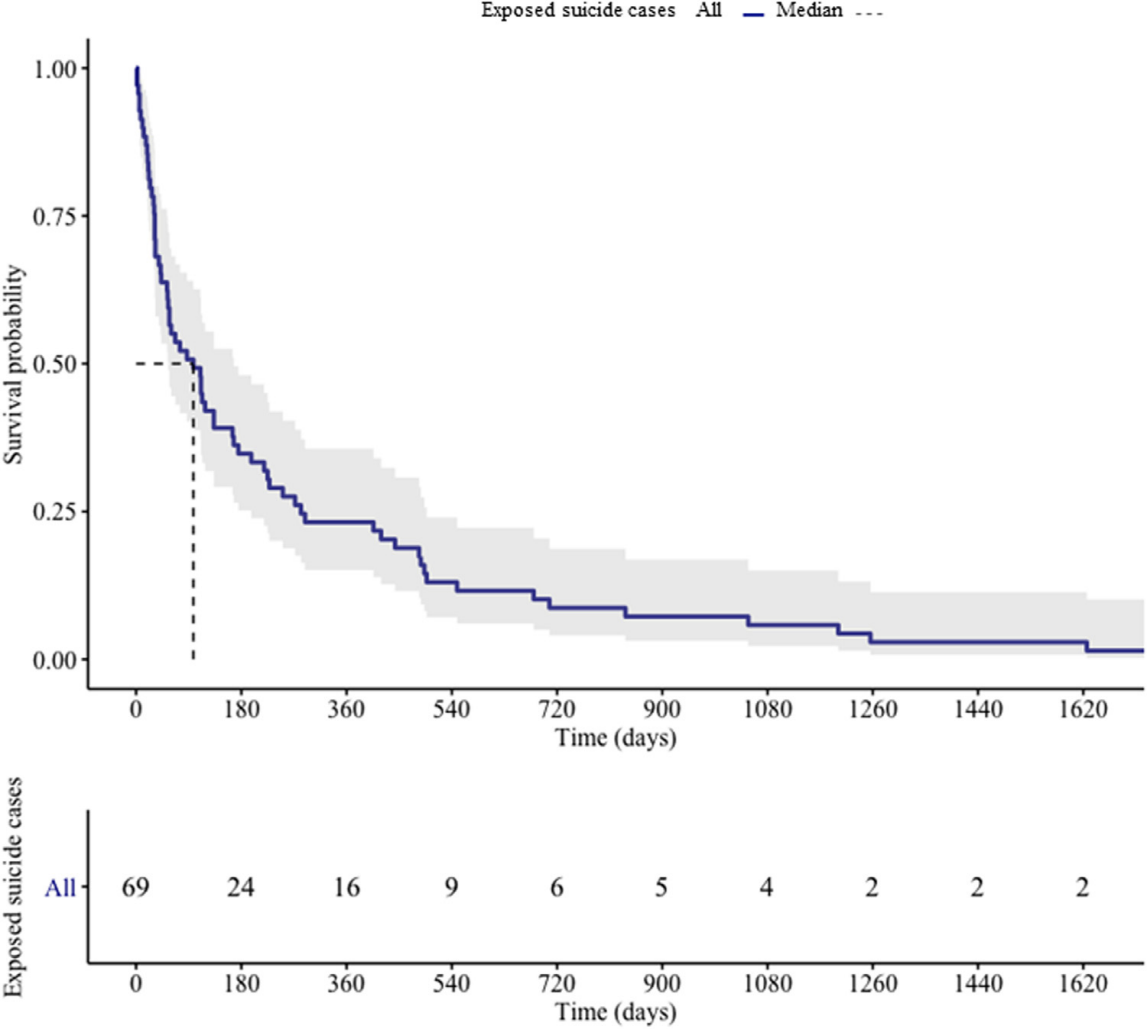
Survival time among exposed cases estimated by the Kaplan Meier method. The point on the x-axis where the horizontal dashed line at a survival probability of 0.50 intersects with the curve represents the estimated median survival time of exposed cases.

The mean age of the 117 young people who died by suicide and could be linked to the suicide of another young person was 18.7 years (SD=3·1, range= 10–24 years). The majority of linked cases were friends and acquaintances (53/69, 77%), and (19/69, 23%) were exposed to both a relative and friend or acquaintance. The demographic, social, and clinical variables as well as the circumstances of death among index and exposed cases are shown in Table 2. Overall, 33 (55%) index and exposed cases lived in SA2s that were within a 10 km of one another and an additional 19 (37%) lived within 100 km. Combined, more than two thirds (75%) of index and exposed cases lived in SA2s that were separated by less than 100kms (Fig. 2). Post hoc analysis of the correlation between distance and time was small and non-significant (*r* = 0.1, 95% CI= 0·1 to 0·4) suggesting that individuals did not relocate over longer time periods. Fig. 3 shows the number of incident suicide cluster events and cluster members that occurred each year, throughout the study period. Between 2006 and 2015 the number of suicide cluster events increased by an annual average percent change of 50%. Results of the survival analysis revealed that the median duration of survival among exposed cases was 87 days (Fig. 4). In other words, 50% of exposed cases died within 90 days of exposure to an index suicide (range = 2 to 2814 days, interquartile range= 250 days).

**Table 2:** Demographic, social, clinical risk factors and circumstances of death amongst index and exposed cases.

Variable	Total (*N* = 117)		Index Cases (*N* = 55)		Exposed Cases (*N* = 62)		*X^2^ P* value
	N (%)		N (%)		N (%)		
Demographic characteristics							
Sex	77	(65.81)	37	(67.27)	40	(64.52)	0.905
Aged 18 years or less	57	(48.72)	24	(43.64)	33	(53.23)	0.395
Aboriginal or Torres Strait Island origins	27	(23.08)	13	(23.64)	14	(22.58)	1
Employed	37	(31.62)	18	(32.73)	19	(30.65)	0.966
Student	38	(32.48)	17	(30.91)	21	(33.9)	0.885
Socioeconomic advantage and disadvantage (SEIFA)^1^	40	(34.19)	20	(36.36)	20	(32.26)	0.785
Residing in a remote location	15	(12.82)	7	(12.73)	8	(12.90)	1
Parent separation	39	(33.33)	12	(21.82)	27	(43.55)	0.022
Living alone at time of death	<10	(<8.5)	<5	(<9.1)	<5	(<8.1)	1
Social characteristics							
Relationship breakdown (past month)	23	(19.66)	11	(20.0)	12	(19.35)	1
Peer conflict (past year)	<10	(<8.5)	<5	(<9.1)	6	(9.68)	0.279
Family conflict	24	(20.51)	11	(20.0)	13	(20.97)	1
Binge drinking (past month)	23	(19.66)	14	(25.45)	9	(14.52)	0.21
History of abuse or neglect	<10	(<8.5)	<5	(<9.1)	12	(19.35)	0.02
Exposure to Domestic violence	<10	(<8.5)	<5	(<9.1)	5	(8.1)	0.06
Clinical characteristics							
Diagnosed or undiagnosed mental illness	62	(53.0)	35	(63.64)	27	(43.55)	0.541
Self-harm (past year)	<10	(<8.5)	<5	(<9.1)	<5	(<8.1)	0.871
Discharged from the ED^2^	10	(8.55)	5	(9.1)	5	(8.1)	1
Illicit substances	35	(29.91)	20	(36.36)	15	(24.19)	0.218
Alcohol impairment	14	(12.0)	8	(14.55)	6	(9.68)	0.6
Circumstances of death							
Suicide note available	33	(28.21)	15	(27.27)	18	(29.03)	0.413
Told someone they wanted to die	49	(41.90)	19	(34.55)	30	(48.39)	0.184
Communicated on social media	<10	(<8.5)	<5	(<9.1)	5	(8.1)	0.265
Internet search for methods	<10	(<8.5)	<5	(<9.1)	<5	(<8.1)	1

1 Socioeconomic Index for Advantage and Disadvantage bottom 20th percentile for relative disadvantage based on SA2 for place of residence

2 ED= Emergency department Counts less than *N* = 5 were aggregated to preserve anonymity.

### 3.3 Investigation of assortative relating

**Fig. 5:**
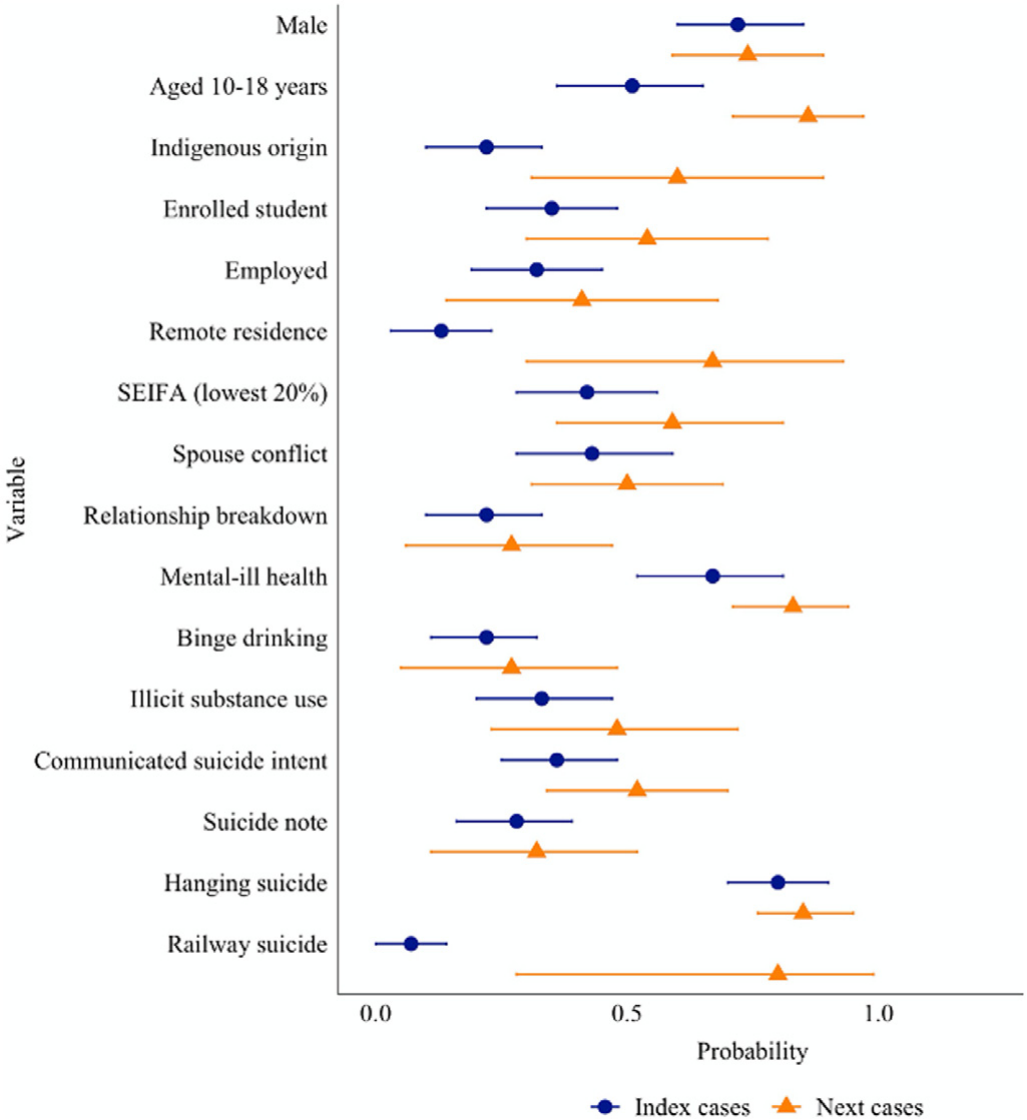
Casewise concordance of riskfactors among index and exposed cases.

Fig. 5 presents the conditional probability that the exposed case had the same characteristic, given the exposure status of the index case. In 86% of cases (95% CI 0·7 to 1·0), the exposed case was also aged 18 years or less when the index case was in this age group. The probability that a young person was of Aboriginal and Torres Strait Island descent was 60% (95% CI= 0·3 to 0·9), when the index case was also of Aboriginal and Torres Strait Island descent. For young people living in remote or very remote locations, the probability in that an exposed case was also from a remote location was 67% (95% CI= 0·3 to 0·9). No further differences were observed between index and exposed cases for history of mental-ill health (83%, 95% CI= 0.71 to 0.94), illicit substance misuse (23%, 95% CI= 0.23 to 0.72), history of binge drinking (27%, 95% CI= 0.05 to 0.48) and the remaining demographic, social and clinical characteristics (Table 3).

**Table 3:** Results of the casewise concordance among index and exposed cases who died in suicide clusters.

	N exposed	N observations	Probability	95% CI Lower	95% CI Upper
Male index case	50	69	0.72	0.60	0.85
Male exposed case	37	50	0.74	0.59	0.89
Aged 10–18 years index case	35	69	0.51	0.36	0.65
Aged 10–18 years exposed case	30	35	0.86	0.71	1.00
Aboriginal and Torres Strait Island origin index case	15	69	0.22	0.10	0.33
Aboriginal and Torres Strait Istland origin exposed case	9	15	0.60	0.31	0.89
Enrolled student index case	24	69	0.35	0.22	0.48
Enrolled student index case	13	24	0.54	0.30	0.78
Employed index case	22	69	0.32	0.19	0.45
Employed exposed case	9	22	0.41	0.14	0.68
Residence in remote region index case	9	69	0.13	0.03	0.23
Residence in remote region exposed case	6	9	0.67	0.30	0.93
SEIFA (Bottom 20%) index case	29	69	0.42	0.28	0.56
SEIFA (Bottom 20%) exposed case	17	29	0.59	0.36	0.81
Conflict with spouse (past month) index case	30	69	0.43	0.28	0.59
Conflict with spouse (past month) index case	15	30	0.50	0.31	0.69
Relationship breakdown (past month) index case	15	69	0.22	0.10	0.33
Relationship breakdown (past month) index case	4	15	0.27	0.06	0.47
Mental ill health index case	46	69	0.67	0.52	0.81
Mental ill health exposed case	38	46	0.83	0.71	0.94
History of binge drinking index case	15	69	0.22	0.11	0.32
History of binge drinking exposed case	4	15	0.27	0.05	0.48
Illicit substances detected in index case	23	69	0.33	0.20	0.47
Illicit substances detected in exposed case	11	23	0.48	0.23	0.72
Told someone they wanted to die by suicide index case	25	69	0.36	0.25	0.48
Told someone they wanted to die by suicide exposed case	13	25	0.52	0.34	0.70
Suicide note index case	19	69	0.28	0.16	0.39
Suicide note exposed case	6	19	0.32	0.11	0.52
Family conflict (past year) index case	13	69	0.19	0.08	0.30
Family conflict (past year) exposed case	2	13	0.02	0.45	0.35
**Methods**					
Hanging index	55	69	0.80	0.70	0.90
Hanging exposed case	47	55	0.85	0.76	0.95
Railway index	5	69	0.07	0.00	0.14
Railway exposed case	4	5	0.80	0.28	0.99

SEIFA= Socio Economic Index for advantage and disadvantage (bottom 20th percentile) based on SA2 area of residence.

### 3.4 Investigation into the social transmission of suicidal behaviour

Results of the casewise concordance analyses showed that exposed cases had an 80% (95% CI = 0·3 to 1·0) probability of dying by railway suicide when exposed to an index suicide that died by the same method (Fig. 5). The conditional probability of additional methods (other than hanging) was not estimated due to insufficient numbers. No significant differences in probability were observed across the remaining characteristics (Table 3).

## 4 Discussion

This study investigated characteristics and circumstances of death of 117 young people aged 10–24 years who died in suicide clusters in Australia in 2006–2015. Results from the present study found evidence of both the social transmission of suicidal behaviour and assortative relating. In particular, by examining the concordance of demographic, social and clinical characteristics of index and exposed cases in a cluster, we found that the presence of pre-existing clinical characteristics such as mental-ill health, substance misuse, and history of binge drinking among index cases were not significantly associated with the probability that the same characteristics were present among exposed cases. Instead, young people who reside in remote communities and were of Aboriginal and Torres Strait Island background had a significant probability of being involved in a suicide cluster, when exposed to an index suicide of the same origins. Together these findings provide some evidence of assortative relating, but these findings were most robust in young people under the age of 18, those who were of Aboriginal and Torres Strait Island descent, and those resided in a remote location. Additionally, results from the present study provide indicative support for the role of the social transmission of suicidal behaviour. In particular, young people who were exposed to the railway suicide of a peer had an 80% probability of dying by the same method. Given suicide by railway is a particularly rare and violent method, it is plausible that the social transmission of suicidal behaviour, facilitated by knowledge of the index suicide, accounted for this finding.

Results from the concordance analyses revealed that in nearly 80% of cases members of suicide clusters belonged to the same age group as the index suicide. These findings suggest that the younger cohort may be particularly susceptible to suicide following exposure to the suicide of a friend or acquaintance. Additionally, we found that 50% of exposed cases died within 90 days of exposure to an index suicide. Together, these findings suggest that the first three months of following exposure to the suicide of another young person may represent a period of heightened suicide risk and therefore represents a critical window of opportunity for the postvention and cluster response interventions with the aim of averting further suicide deaths.

Identifying the risk factors associated with suicide clusters has the potential to inform targeted cluster response strategies, with better precision. The finding that young people who reside in remote communities may be particularly vulnerable following exposure to the suicide of a peer suggests that remote communities should be particularly vigilant following a suicide in the community. For example, remote communities may benefit from developing a postvention protocol that ensures members of the community have adequate access to postvention support following a suicide. Furthermore, remote communities may benefit from universal suicide prevention interventions that equip gatekeepers to respond to those who may be at risk of suicide, particularly following a suicide in the community.

It is noteworthy that we found limited concordance between index and exposed cases based on clinical risk factors such as history of adverse mental health. Screening for suicide in high risk populations is a core component of existing cluster response guidelines [12–14,30], despite evidence that suggests that screening for suicide risk is associated with a positive predictive value of approximately 6% [31]. Instead, results of the present study suggest that the presence of pre-existing clinical risk factors may be less sensitive predictors of young people who are at risk of suicidal behaviour who are later involved in suicide clusters. Based on these findings, we recommend that, in addition identifying young people experiencing significant distress, screening efforts should be broadened to include identifying individuals who were friends of the deceased regardless of their pre-existing vulnerabilities, and when necessary, these young people should be provided with access to postvention support.

Further, since 50% of exposed cases died within the first 90 days of exposure, repeat screening during this period may be particularly warranted. It is equally important that communities are equipped with resources to effectively mitigate the social transmission of suicidal behaviour. To date, there is a paucity of studies that have investigated the effectiveness of interventions on preventing the social transmission of suicidal behaviour in young people [32]. The evaluation of community-based interventions in response to suicide clusters as well as the development of targeted interventions is essential to fill these critical gaps in evidence.

Although geographic proximity appears to be an important characteristic of the social transmission of suicidal behaviour, it is noteworthy that approximately 25% of index and exposed cases lived in regions that were separated by at least 100 km in distance. The distance between index and exposed cases was poorly correlated with time, suggesting that the distance between index and exposed cases did not increase over time (i.e., because of the relocation of exposed cases). One explanation is that the popular and widespread use of social networking and other digital media influences may facilitate the spread of suicidal behaviour among index and exposed cases [2]. A study by Robertson and colleagues, investigated the social links between members of suicide cluster in a small rural community in New Zealand. By combining both an inferential and descriptive case study approach, the authors identified additional members who were linked to the suicide cluster via social media but did not meet the authors criteria for geographic proximity using statistical analyses alone [33].

Communication via the internet and social media is well ingrained in the lives of young people and although there are many benefits, there is a need to further equip young people to safely communicate about suicide online [34]. This is particularly important given communication via the internet and social media has the potential to transcend traditional geographic boundaries associated with the development and maintenance of suicide clusters [2]. Whilst existing frameworks for the detection, response and prevention of suicide clusters provide recommendations for safe reporting of suicide in the traditional media, communities may also benefit from the implementation of interventions that equip young people to communicate about suicide in online environments in a safe way (e.g., without interfering with the grieving process).

Although the NCIS offers the best available information on suicide and external causes of death in Australia, the quality of data in the NCIS vary considerably between individual cases and coronial jurisdictions. Information on exposure to suicide and suicide characteristics was only available for analysis if it was queried during the formal police or coroner investigation and reported by informants during the investigation. This resulted in the exclusion of 49 exposed cases that did not provide sufficient information on the index suicide. Furthermore, variables which were not reported in the police or coroner records were coded as not being present. Consequently, there was likely to be some misclassification bias, where absence of evidence was treated as evidence of absence. The limitations associated with the completeness and quality of the data, and the retrospective nature of the study, meant that the operationalisation of the social transmission of suicidal behaviour and assortative relating were limited to the variables reported in the NCIS and may therefore lack precision. For example, due to power limitations it was not possible to examine the concordance between other methods of suicide such as poisoning by gaseous substances that have been reported in previous reports of suicide clusters [35].

Our analysis of cluster-linked pairs was limited to young people aged 24 year or less who died by suicide during the study period. This resulted in the exclusion of 60 (29.7%) index cases over the age of 25. Given exposure to the suicide of an adult accounted for one third of exposure events, future studies ought to consider examining the characteristics associated with exposure to suicide across the lifespan. Additionally, due to the absence of existing methodologies to control for multiple dependencies in casewise analyses, it was not possible to determine concordance rates among index and exposed cases using multivariate analyses. Future studies which overcome these limitations in order to better understand the interaction between covariates in casewise analysis is therefore warranted.

Lastly, results of the casewise analysis revealed that young people who were exposed to the suicide of a peer who resided in a remote community or had Indigenous heritage were likely to reside in the same community settings. Evidence of suicide clusters occurring in remote and indigenous communities is widely documented in both descriptive case studies and population-based studies using data from national suicide registries [6,7]. However it is not clear whether these findings are consistent with the social transmission of suicidal behaviour or assortative relating hypothesis. It is possible that environments that are characterised by high levels of social cohesion enable the rapid spread of information between members of the community [2,36]. For example, Important cultural practices and obligations around death, also known as sorry business, are experienced by direct and extended kinship networks and it is not uncommon for members of the clan to travel hundreds of kilometres to attend sorry business ceremonies [37,38]. Future studies which investigate the mechanisms associated with suicide clusters in specific high-risk communities may provide important insight into these existing limitations.

Although young people involved in suicide clusters may be susceptible to a number of demographic, social and clinical risk factors, sharing these factors does not necessarily account for the association between index and exposed cases involved in suicide clusters. Instead we found that the circumstances of death and social factors such as age of the deceased, remoteness of residence and Aboriginal and Torres Strait Islander heritage may be of equal importance for the prevention of youth suicide clusters. Findings from the present study have important implications for our understanding of who might be at risk of suicide following exposure to the suicide of a friend or acquaintance and provides important insight into potential strategies for preventing and responding to suicide clusters. In particular, we recommend that, in addition to screening for suicide risk, individuals who were close to the deceased should be provided with access to postvention support and that interventions targeting young people at risk of being involved in a suicide cluster may be particularly beneficial within the first 90 days following a suicide in the community.

## Funding

NTMH is a PhD student and was supported by the Australian Rotary Health PhD Partnership Scholarship. MS is supported by an Australian Research Council Future Fellowship (grant number FT180100075). JP is supported by a National Health and Medical Research Council (NHMRC) Investigator Grant (grant number GNT1173126). MT is supported by a National Health and Medical Research Council (NHMRC) Early Career Fellowship (grant number GNT1138710). JR is supported by a National Health and Medical Research Council (NHMRC) Career Development Fellowship (grant number GNT1142348). The funders of the study had no role in study design, data collection, data analysis, data interpretation, or writing of the report. The corresponding author had full access to all the data in the study and had final responsibility for the decision to submit for publication.

## Data sharing statement

All data reported in this study was accessed through the National Coronial Information System by NTMH. The database used in the current study is not publicly available due to the sensitivity of the suicide data. Access requests can be made directly by submitting an application to the National Coronial Information System.

## Author contributions

NTMH was responsible for the study design, data collection, data analysis, data interpretation, drafting the manuscript and revising the manuscript. MS was responsible for the study design, data analysis, drafting the manuscript and revising the manuscript. JP was responsible for data interpretation and writing the initial manuscript. MT was responsible for writing the initial manuscript. JR was responsible for writing the initial manuscript.

## Declaration of Competing Interest

The authors declare no competing interests.

## Appendix Supplementary materials

Image, application 1

## References

[1] World Health Organization (2016). Number of suicides globally in young people. http://www.who.int/healthinfo/global_burden_disease/estimates.

[2] Hawton K., Hill N.T.M., Gould M., John A., Lascelles K., Robinson J (2019). Clustering of suicides in children and adolescents. Lancet Child Adolesc Health.

[3] Hawton K., Saunders K.E., O'Connor R.C (2012). Self-harm and suicide in adolescents. Lancet.

[4] Robinson J., Too L.S., Pirkis J., Spittal M.J (2016). Spatial suicide clusters in Australia between 2010 and 2012: a comparison of cluster and non-cluster among young people and adults. BMC Psychiatry.

[5] Gould M.S., Wallenstein S., Kleinman M (1990). Time-space clustering of teenage suicide. Am. J. Epidemiol..

[6] Haw C., Hawton K., Niedzwiedz C., Platt S (2013). Suicide clusters: a review of risk factors and mechanisms. Suicid Life Threat Behav.

[7] Niedzwiedz C., Haw C., Hawton K., Platt S (2014). The definition and epidemiology of clusters of suicidal behavior: a systematic review. Suicid Life Threat Behav.

[8] Hill N.T.M., Robinson J., Pirkis J., Andriessen K., Krysinska K., Payne A. (2020). Association of suicidal behavior with exposure to suicide and suicide attempt: a systematic review and multilevel meta-analysis. PLoS Med..

[9] Callahan J. (1996). Negative effects of a school suicide postvention program–a case example. Crisis.

[10] Heffel C.J., Riggs S.A., Ruiz J.M., Ruggles M (2015). The aftermath of a suicide cluster in the age of online social networking: a qualitative analysis of adolescent grief reactions. Contemp Sch Psychol.

[11] Abbott C.H., Zakriski A.L. (2014). Grief and attitudes toward suicide in peers affected by a cluster of suicides as adolescents. Suicid Life-Threat Behav.

[12] Palmer S., Inder M., Shave R., Bushnell J (2018). Postvention guidelines for the management of suicide clusters. Clin Adv Serv Aotearoa.

[13] Health Do, Public Health England (2015). Identifying and responding to suicide clusters and contagion: a practice resource.

[14] Public Health England (2019). Identifying and responding to suicide clusters: a practice resource.

[15] Witt K., Robinson J. (2019). Sentinel surveillance for self-harm. Crisis.

[16] Reyes-Portillo J.A., Lake A.M., Kleinman M., Gould M.S (2019). The relation between descriptive norms, suicide ideation, and suicide attempts among adolescents. Suicide Life Threat Behav.

[17] Hawton K., Hill N.T.M., Gould M., John A., Lascelles K., Robinson J (2020). Clustering of suicides in children and adolescents. Lancet Child Adolesc Health.

[18] Gould M.S., Kleinman M.H., Lake A.M., Forman J., Midle J.B (2014). Newspaper coverage of suicide and initiation of suicide clusters in teenagers in the USA, 1988–96: a retrospective, population-based, case-control study. Lancet Psychiatry.

[19] Joiner T.E. (1999). The clustering and contagion of suicide. Curr Dir Psychol Sci.

[20] Schaefer D.R., Kornienko O., Fox A.M (2011). Misery does not love company: network selection mechanisms and depression homophily. Am Sociol Rev.

[21] Cairns R.B., Cairns B.D., Neckerman H.J., Gest S.D., Gariepy J.-.L (1988). Social networks and aggressive behavior: peer support or peer rejection. Dev Psychol.

[22] Davidson L.E., Rosenberg M.L., Mercy J.A., Franklin J., Simmons J.T (1989). An epidemiologic study of risk factors in two teenage suicide clusters. JAMA.

[23] Brent D.A., Kerr M.M., Goldstein C., Bozigar J., Wartella M., Allan M.J (1989). An outbreak of suicide and suicidal behavior in a high school. J. Am. Acad. Child Adolesc. Psychiatry.

[24] Durkheim E. (1951). Suicide.

[25] Mueller A.S., Abrutyn S (2016). Adolescents under pressure: a new Durkheimian framework for understanding adolescent suicide in a cohesive community. Am Sociol Rev.

[26] Hill N.T.M., Too L.S., Spittal M.J., Robinson J (2020). Understanding the characteristics and mechanisms underlying suicide clusters in Australian youth: a comparison of cluster detection methods. Epidemiol Psychiatr Sci.

[27] Bugeja L., Clapperton A.J., Killian J.J., Stephan K.L., Ozanne-Smith J (2010). Reliability of ICD-10 external cause of death codes in the National Coroners Information System. Health Inf Manag J.

[28] Daking L., Dodds L. (2007). ICD-10 mortality coding and the NCIS: a comparative study. Health Inf Manag J.

[29] Witte J.S., Carlin J.B., Hopper J.L (1999). Likelihood-based approach to estimating twin concordance for dichotomous traits. Genet Epidemiol.

[30] Centers for Disease Control (1988). CDC recommendations for a community plan for the prevention and containment of suicide clusters. Morb Mortal Wkly Rep.

[31] Carter G., Milner A., McGill K., Pirkis J., Kapur N., Spittal M.J (2017). Predicting suicidal behaviours using clinical instruments: systematic review and meta-analysis of positive predictive values for risk scales. Br J Psychiatry.

[32] Cox G.R., Robinson J., Williamson M., Lockley A., Cheung Y.T., Pirkis J (2012). Suicide clusters in young people: evidence for the effectiveness of postvention strategies. Crisis.

[33] Robertson L., Skegg K., Poore M., Williams S., Taylor B (2012). An adolescent suicide cluster and the possible role of electronic communication technology. Crisis.

[34] Robinson J., Hill N.T.M., Thorn P., Battersby R., Teh Z., Reavley N.J. (2018). The #chatsafe project. Developing guidelines to help young people communicate safely about suicide on social media: a Delphi study. PLoS ONE.

[35] Centres for Disease Control. Cluster of suicides and suicide attempts. New Jersey; 1988.

[36] Hanssens L. (2008). Imitation and contagion contributing to suicide clustering in indigenous communities: time-space-method cluster analysis. Aborig Isl Health Work J.

[37] Carlson B., Frazer R. (2015). It's like going to a cemetery and lighting a candle”: aboriginal Australians, sorry business and social media. AlterNative: Int J Indig Peoples.

[38] Hanssens L. (2008). Clusters of suicide the need for a comprehensive postvention response to sorrow in indigenous communities in the northern territory. Aborig Isl Health Work J.

