# Supplementary material for: Risk factors associated with suicide clusters in Australian youth: Identifying who is at risk and the mechanisms associated with cluster membership
Source: eClinicalMedicine. 2020 Nov 20;29-30:100631. doi: 10.1016/j.eclinm.2020.100631 (PMC7691728; doi:10.1016/j.eclinm.2020.100631)
Supplement: Supplementary file 1 [file mmc1.docx]

# Appendix

**Table A1. Pair configurations and dependencies among suicide descendants involved in suicide clusters**


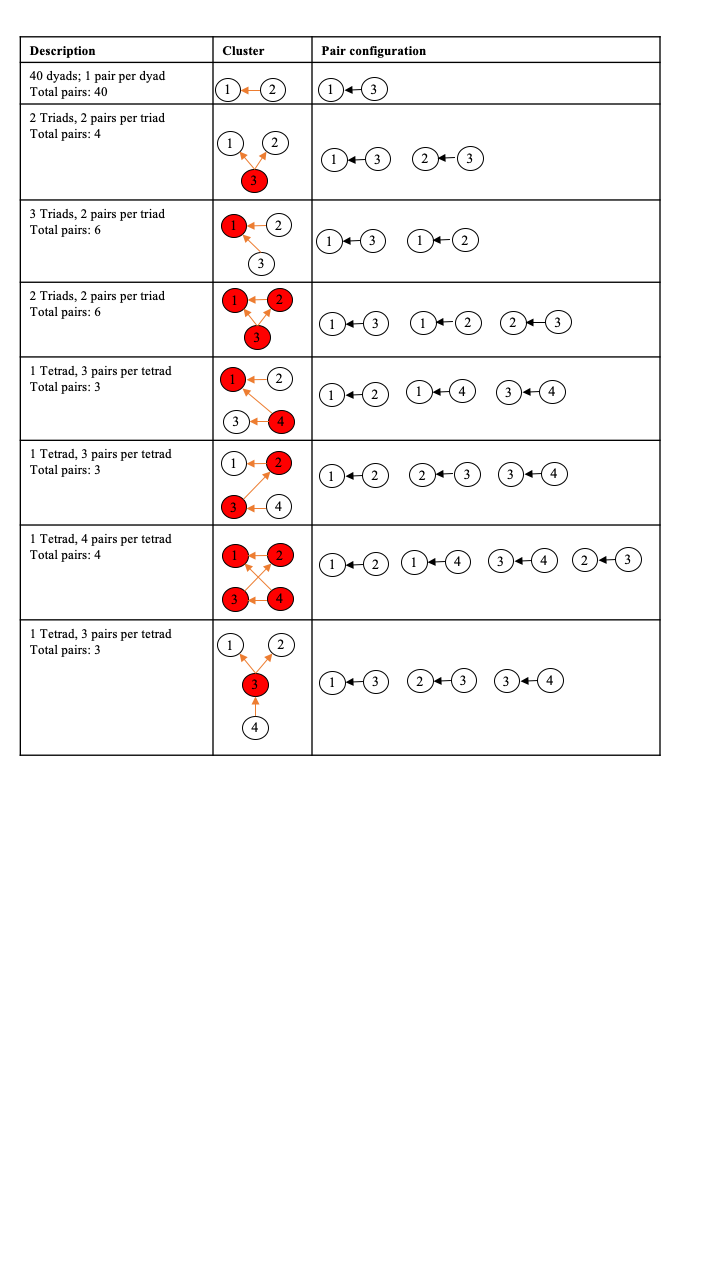


The numbers represent the sequence of death and the arrows represent the direction of exposure. Red circles represent dependencies within clusters (suicides that were both an index and exposed case AND/OR were included in more than one pair).

**Analysis of missing cases**

We could not identify reliable links among 49 young people (40.8%) who were exposed to suicide during the study period. This was because there was insufficient information on the index case, the index case could not be identified based on the information available (the person or date of death was not recorded in the NCIS), or the index case died prior to the beginning of the study period. However, an analysis of the NCIS core variables indicated that exposed cases that could and could not be linked to an index case were comparable across the full range of demographic variables (i.e., age, sex, Aboriginal and Torres Strait Islander status, employment status, student status, residential remoteness) recorded in the NCIS (table A2).

**Table A2. Characteristics of linked and missing cases in the NCIS**

| **Variable** |  | **Eligible for linkage**  **N= 69** | | **Missing Information**  **N=49** | | **X^2^ *P* value** | | |
| --- | --- | --- | --- | --- | --- | --- | --- | --- |
|  |  | N | % | N | % |  | |  |
| Sex (male) |  | 44 | 63.8 | 35 | 71.4 |  | 0.501 | |
| Aged 18 years or less | | 35 | 50.7 | 22 | 44.9 |  | 0.662 | |
| Aboriginal or Torres Strait Island origins | | 15 | 21.7 | 9 | 18.4 |  | 0.828 | |
| Employed status |  | 22 | 31.9 | 16 | 32.7 |  | 1.000 | |
| Student status |  | 23 | 33.3 | 19 | 38.8 |  | 0.679 | |
| Residing in a remote location | | 9 | 13.0 | <5 | <10.2 |  | 0.354^1^ | |
| SEIFA^2^ |  | 22 | 31.9 | 10 | 20.4 |  | 0.241 | |

^1^ Fishers exact test

^2^ SEIFA= Socioeconomic Index for Advantage and Disadvantage bottom 20^th^ percentile for relative disadvantage based on SA2 for place of residence.
